# Enhanced Particulate Organic Carbon Export at Eddy Edges in the Oligotrophic Western North Pacific Ocean

**DOI:** 10.1371/journal.pone.0131538

**Published:** 2015-07-14

**Authors:** Yung-Yen Shih, Chin-Chang Hung, Gwo-Ching Gong, Wan-Chen Chung, Yu-Huai Wang, I-Huan Lee, Kuo-Shu Chen, Chuang-Yi Ho

**Affiliations:** 1 Department of Oceanography, National Sun Yat-sen University, Kaohsiung, 80424, Taiwan; 2 Institute of Marine Environmental Chemistry and Ecology, National Taiwan Ocean University, Keelung, 20224, Taiwan; CAS, CHINA

## Abstract

Mesoscale eddies in the subtropical oligotrophic ocean are ubiquitous and play an important role in nutrient supply and oceanic primary production. However, it is still unclear whether these mesoscale eddies can efficiently transfer CO_2_ from the atmosphere to deep waters via biological pump because of the sampling difficulty due to their transient nature. In 2007, particulate organic carbon (POC) fluxes, measured below the euphotic zone at the edge of warm eddy were 136–194 mg-C m^−2^ d^−1^ which was greatly elevated over that (POC flux = 26–35 mg-C m^−2^ d^−1^) determined in the nutrient-depleted oligotrophic waters in the Western North Pacific (WNP). In 2010, higher POC fluxes (83–115 mg-C m^−2^ d^−1^) were also observed at the boundary of mesoscale eddies in the WNP. The enhanced POC flux at the edge of eddies was mainly attributed to both large denuded diatom frustules and zooplankton fecal pellets based on scanning electron microscopy (SEM) examination. The result suggests that mesoscale eddies in the oligotrophic waters in the subtropical WNP can efficiently increase the oceanic carbon export flux and the eddy edge is a crucial conduit in carbon sequestration to deep waters.

## Introduction

The oligotrophic open waters compose of the major part (~75%) of the surface ocean and account for over 30% of the global marine carbon fixation [1]. The oligotrophic waters can sequestrate atmospheric CO_2_ to the ocean interior via physical and biological pumps [2] because of the important contribution of these oligotrophic waters to global carbon cycling. The subtropical oligotrophic Western North Pacific (NWP) Ocean is a nitrate-deficient water, where nitrate is usually undetectable (< 0.1 μM) in the upper layer based on year-round investigations [3,4]. This region is also characterized by low-chlorophyll-*a* (Chl *a*) concentration exhibiting unremarkable seasonal variability [3,4]. *Synechococcus* and *Prochlorococcus* are the main phytoplankton groups in the oligotrophic NWP [5–7]. The particulate organic carbon (POC) flux is not high (20~30 mg-C m^−2^ d^−1^) based on four-season investigations and does not show strong seasonal variations except for extreme weather events (typhoon and Asian dust storm) [4,8,9].

Mesoscale eddies are frequently observed in the oligotrophic ocean, and may play a crucial role in transporting energy, heat, biological assemblages, nutrients and stimulating phytoplankton blooms [10–12]. In general, cold eddies contain upwelling nutrient-rich waters, thus resulting in higher phytoplankton biomass and primary productivity (PP). Warm eddies normally contain nutrient-depleted waters with lower phytoplankton biomass and lower PP [13–18]. However, there are inconsistent reports about organic carbon flux in different eddy systems. For example, Hung et al. (2004) [19] measured organic carbon fluxes (herein POC flux measured at depth of 120 m) in cold and warm eddies in the oligotrophic water of Gulf of Mexico and found cold eddies with elevated organic carbon flux and warm eddies with low organic carbon flux (Table 1) in 2000, but organic carbon flux did not show remarkable difference in the cold eddy center and a warm eddy in 2001.

**Table 1:** POC export fluxes related to eddies that determined by different methods and surveyed in different areas.

Locations	Depth(m)	POC flux(mg-c m^-2^ day^-1^)		Method	Reference
GOM(year: 2000)	120	148(C)	60(W)	Trap	Hung et al. 2004
GOM(year: 2001)	120	48(C)	39(W)	Trap	Hung et al. 2004
GOM	120	24–71(C)	58–94(W)	Trap	Hung et al. 2010
lee of Hawaii	150	31.2(inside C)	12.2(outside C)	^234^Th*	Bidigare et al. 2003
lee of Hawaii	150	18.5(inside C)	18.2(outside C)	Trap	Benitez-Nelson et al. 2007
lee of Hawaii	150	19–21(inside C)	20(outside C)	^210^Pb*	Verdeny et al. 2008
BATS	150	20.4(C)	16.8(M)	Trap &^234^Th*	Buesseler et al. 2008
SCS	125	72.6(C)	35.9(W)	Model	Xiu and Chai 2011
North SCS	100	45.4(W core)	41.6(W edge)	^234^Th*	Zhou et al. 2013

GOM: Gulf of Mexico; BATS: Bermuda Atlantic Time-series Study; SCS: South China Sea; C: cold eddy; W: warm eddy; M: mode water

*: methods of radionuclide disequilibrium (^234^Th/^238^U, ^210^Pb/^210^P_o_; particle size >53μm).

Some researchers reported that low carbon export fluxes were found inside or outside of mesoscale eddies either in the lee of Hawaii or in BATS (Bermuda Atlantic Time-series Study) station (Table 1) [20–23]. Hung et al. (2010a) [24] reported elevated POC flux on the northern edge of a warm eddy and low POC flux inside cold eddy in oligotrophic water of Gulf of Mexico. Zhou et al. (2013) [25] estimated organic carbon flux in the South China Sea and suggested that high POC export can be found at the eddy edge rather than inside of eddy (Table 1). Benitez-Nelson et al. (2007) [21] measured POC flux and other biogeochemical parameters within a cold eddy off Hawaii in warm Pacific Ocean and concluded that elevated primary production and community biomass were found within a cold eddy, but had little influence on organic carbon export in the warm waters of the Pacific Ocean based on one single eddy.

According to these inconsistent reports, it can be summarized two key points: (1) cold eddy indeed can bring nutrient to the euphotic zone and then stimulate phytoplankton growth (i.e. PP), but it is still unclear whether mesoscale eddies can efficiently carry organic carbon into the ocean interior; (2) elevated organic carbon export seems to appear at the eddy edge rather than inside of eddy. As mentioned above, there are numerous mesoscale eddies in the WNP and these eddy edges serve as natural laboratories for better understanding if sinking particles can efficiently transport organic carbon to the euphotic zone in an oligotrophic ocean. In this study, we investigated POC export fluxes at the eddy edge in the WNP in 2007 and 2010 and compared with POC fluxes from different eddy regions.

## Materials and Methods

**Fig 1:**
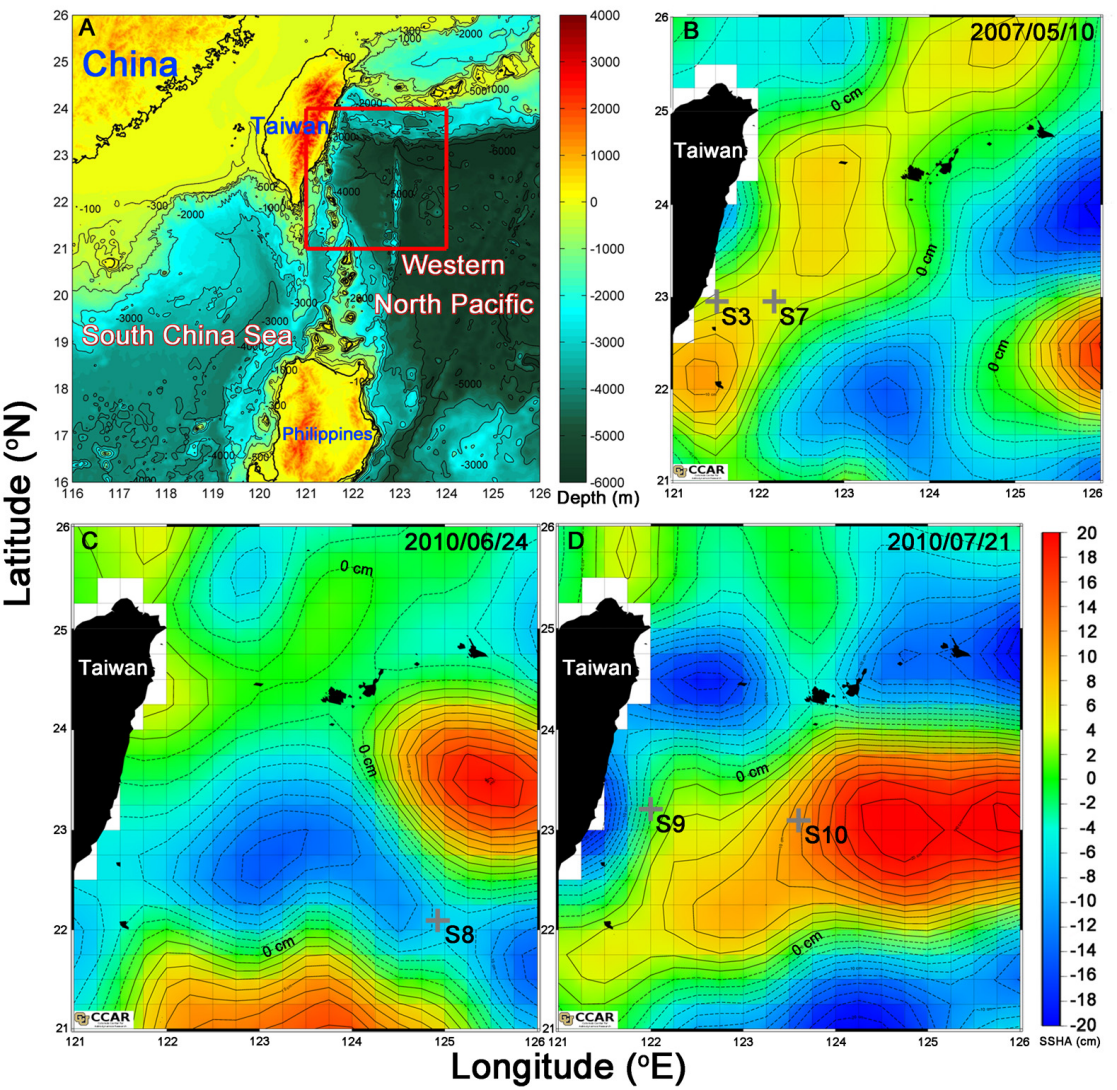
The study area and sampling locations. (A) The main study area on May 10, 2007, June 24 and July 21, 2010, is located in the Western North Pacific (WNP), subtropical oligotrophic ocean basins (red square). The trap deployed stations (cross symbols) in 2007 and 2010 shown as S3 and S7, S8, S9 and S10 were illustrated in the (B)–(D). The sea surface height anomaly (SSHA) maps were derived from CCAR (Colorado Center for Astrodynamics Research). Each interval in the contour (B–D) is 2 cm, solid and dotted lines in the contour depict the positive SSHA (anticyclonic eddies) and negative SSHA (cycloniceddies), respectively.

Seawater samples were collected by a rosette sampler aboard the R/V OR-II in the vicinity of cold and warm eddies in the WNP (Fig 1) during May 9–12, 2007, during June 22–26, 2010 and July 19–22, 2010. No specific permissions were required for these locations. During May cruise in 2007, the survey was conducted along a west—east transect (stations 1–7, S1~S7). Station 3 (S3) (water depth 1556 m, water temperature at 30 m = 26.8°C, as reference station, Table 2) was located within the Kuroshio Current, and station 7 (S7) (water depth 5060 m, water temperature at 30 m = 27.0°C, Table 2) was located on the edge of a warm eddy (Fig 1B). In June 2010, station 8 (S8) was located on the edge of a cold eddy (water depth 5436 m, water temperature at 30 m = 28.1°C, Table 2) (Fig 1C). In July 2010, stations 9 (S9) and 10 (S10) were located on the edges of warm eddy (water depth 3326 m and 6066 m, water temperature at 30 m = 28.8°C and 30.5°C, respectively, Table 2) (Fig 1D). The sea surface height anomaly (SSHA) maps were obtained from the website (http://eddy.colorado.edu/ccar/data_viewer/index) of the CCAR (Colorado Center for Astrodynamics Research). Temperature, salinity and density were recorded by a SeaBird CTD (SBE 9/11). Surface currents at 5 m along transect in 2007 were also obtained by an Acoustic Doppler Current Profiler (ADCP). Concentrations of the nitrate+nitrite (N), phosphate (P), silicate (Si), Chl *a*, POC and PP were measured according to Gong et al. (1999) [3] and Hung et al. (2010b) [26]. The detection limit of N, P, Si and PP and analytical error of POC were 0.3, 0.03, 0.2 μM, 0.05 mg-C m^−3^ h^−1^ and 2–5%, respectively. The 8-days mean PP values was derived from an empirical algorithm by Behrenfeld and Falkowski (1997) [27] (http://www.science.oregonstate.edu/ocean.productivity/index.php).

**Table 2:** Summary informations in this study.

Station	Bottom	SST	T_30m_	SS	SSH	MLD	D_ni_	Z_u_
	m	°C	°C		cm	m	m	m
S1	585	27.4	27.0	34.43	66	27	44	78
S2	446	27.8	27.6	34.46	73	35	8	89
*S3	1556	27.9	26.8	34.48	73	22	27	83
S4	3475	27.4	26.2	34.49	77	10	38	107
S5	4210	27.6	27.3	34.56	84	27	77	112
S6	4673	27.7	27.6	34.51	86	35	105	70
*S7	5060	27.6	27.0	34.51	88	23	60	107
*S8	5436	29.2	28.1	34.34	141	21	101	119
*S9	3326	30.1	28.8	33.96	108	21	95	83
*S10	6066	30.7	30.5	34.54	155	40	126	105

Sampling period: 05/09–05/12, 2007 (S1–S7), 06/22–06/26, 2010 (S8), 07/19–07/22, 2010 (S9–S10); SST: sea surface temperature; T_30m_: seawater temperature at the depth of 30 m; SS: surface salinity; SSH:sea surface height;MLD: mixed layer depth; D_ni_: the depth with (nitrate + nitrite) concentration > 0.3 μM; Z_u_: the depth of euphotic zone, 1% of the surface light intensity;

*: flaoting sediment traps deployed stations.

Sinking particles were collected by the surface buoy-tethered floating intercept sediment trap arrays, which consisted of six cylindrical plastic core tubes (6.8 cm diameter and 1:10 aspect ratio) with honeycomb baffles covering the trap mouths [19] at S3, S7, S8, S9 and S10, respectively (Fig 1B–1D). We also deployed a set of sediment traps with six core tubes: three traps filled with filtered seawater (<0.5 μm) and three filled with brine solutions containing mercuric chloride in the study area according to the method of Wakeham et al. (1997) [28] in order to compare POC fluxes collected by unpoisoned traps to those in poisoned traps. Briefly, sinking particles were filtered to a pre-combusted quartz fiber filter (pore size: 1.0 μm). The trap deployment interval was approximately 24 hours at depths of 90, 120, 140, 150 m and some traps were daily deployed from morning to evening in 2010 due to the overlapped exclusive economic zone between Taiwan and Japan. Large swimmers on the filters were pre-filtered by 330 μm net screen and small swimmers were carefully picked out under a microscope [29,30]. Concentrations of POC in the sinking particles (POC in water column as well) were determined by the CHNS/O Elementar (Vario EL, Germany) and the method of Hung et al. (2012) [31].

Floating sediment traps are directly used to measure POC fluxes in sinking particles, but the results may possibly be biases by hydrodynamic and biological effects [32–34]. However, similar floating sediment traps have been used to measure POC fluxes since 1990 in HOTS (Hawaii Ocean Time-Series) and BATS with minimal biases [29]. Furthermore, POC fluxes in the subtropical oligotrophic regions of the Kuroshio [35], Gulf of Mexico [24] and South China Sea [36] are comparably determined by the ^234^Th/^238^U disequilibrium method and floating sediment traps collecting, suggesting that the values of POC export flux in this study are acceptable. Additionally, Hung et al. (2010b) [26] reported that POC flux in the WNP and found no significant difference in POC flux between night and day. Thus, if diurnal variation in POC flux occurs, this may be minor compared to other possible sources of error in our measurements.

Images of some selected sinking particles were collected from subsamples of bulk sinking particles in the study area. In brief, sinking particles were collected on polycarbonate filters (47 mm, pore size = 0.4μm) and the membrane was washed by Milli-Q water. The membrane was dried and cut into 1-cm circles, and mounted on aluminum stubs. The stubs were coated with gold before scanning electron microscopy (SEM) imaging. The specimens were examined using a Hitachi (S-2400) SEM and clear images of bulk sinking particles were taken directly by SEM [31].

## Results and Discussion

### Hydrographic Data, Primary Production and Particulate Organic Carbon Flux

**Fig 2:**
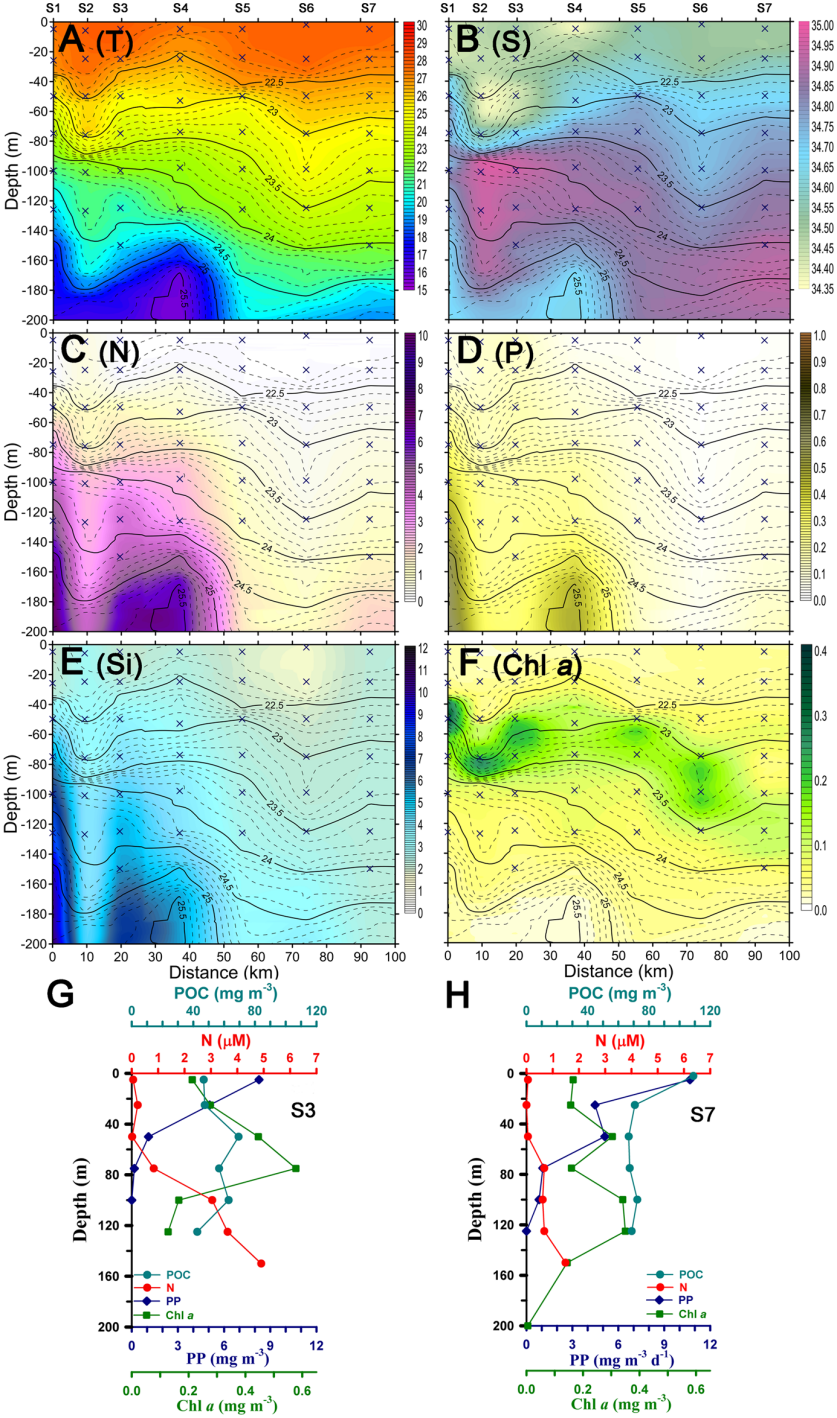
The contoured profiles at S1–S7 and vertical distributions ofbiogeochemi- cal parameters at S3 and S7. Contours of (A) potential temperature (°C), (B) salinity, (C) nitrate + nitrite (N, μM), (D) phosphate (P, μM) (E) silicate (Si, μM) and (F) Chl *a* (mg m^−3^), and vertical distribution of POC, N, Chl *a* and PP at station 3 (S3, G) and 7 (S7, H) in the WNP during cruise in 2007. Vertical cross symbols and lines in the contour depicted depths of seawater measuring and represented isopycnal layer in the upper 200m, respectively.

In 2007, the average sea surface temperature (SST) ranged from 27.4 to 27.9°C at S2~S5 (including reference station S3) was higher than that on the edge of the eddy (i.e. S6–S7, 27.6–27.7°C, Table 2). Although the fact that the depth of the isopycnals at S2–S5 was shallower and narrower than S6–S7, the SSHA and mixed layer depth (MLD, defined as the depth for potential temperature to decrease by 0.8°C from the surface, [37]) showed insignificant variation on both areas (average MLDs were 24, 10–35 and 29, 23–35 m at S2–S5 and S6–S7, respectively) (Fig 2, Table 2) (All relevant data are within the paper and S1 File).

**Fig 3:**
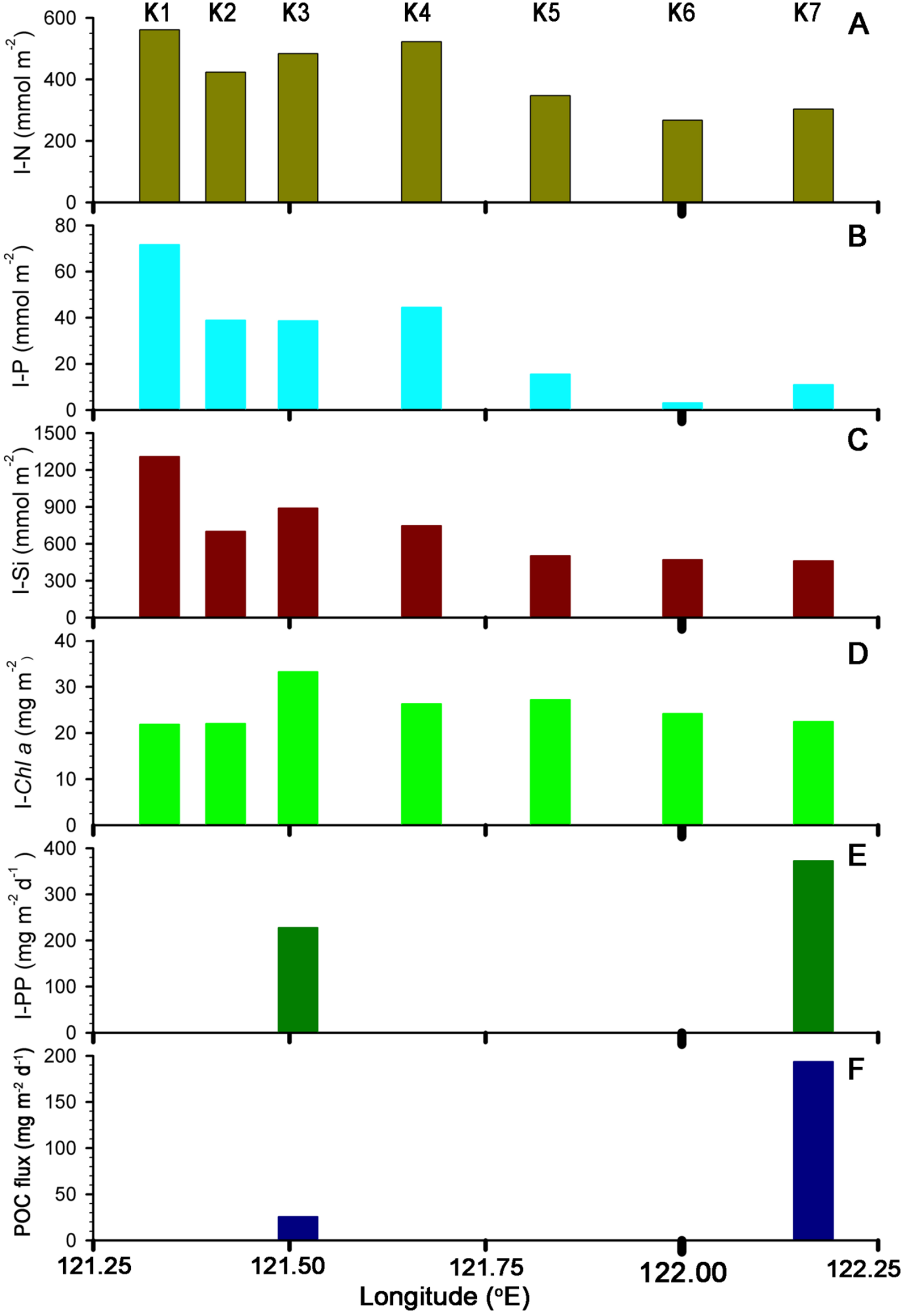
The biogeochemical standing stocks at S1–S7. Sampling locations for (A) nitrate + nitrite (mmol m^−2^), (B) phosphate (mmol m^−2^), (C) silicate (mmol m^−2^) and (D) chl *a* (mg m^−2^) at S1 to S7 (integrated from 0 to 200m); Integrated (E) PP (in-situ incubation primary production, mg m^−2^ d^−1^) from 0 to 125m and (F) POC flux (mg-C m^−2^ d^−1^) at the water depth of 120m at S3 and S7, respectively on May 10, 2007.

Surface nutrient (i.e. N, P, except for Si) concentrations were close to the detection limit among all the stations (Fig 2C and 2D). A substantial amount of nutrients was found within the euphotic zone (the depth defined as 1% of surface light intensity) at S2–S5, while nutrient-rich water was at the bottom of the euphotic zone at S6–S7. Remarkably, the average nitracline depth (D_ni_, defined as the depth with the N concentration >0.3μM between two layers) [38–40] at S2–S5 (38, 8–77 m) was shallower than at S6–S7 (83, 60–105 m) (Table 2). Inventories of N, P and Si (I-N, I-P and I-Si) integrated from 0–200 m were more elevated at S2–S5 (350–520, 20–45 and 500–900 mmol m^−2^) than at S6–S7 (270–300, 3–10 and 460–470 mmol m^−2^) (Fig 3A–3C). Higher nutrient concentrations at S2-S5 can be attributed to the dynamic pumping by the island wake in the main stream of the Kuroshio and a shallower D_ni_ at S2–S5 [35,41].

Low Chl *a* concentrations were found in the surface layer (Fig 2F) and gradually increased to a subsurface Chl *a* maximum (SCM) at most stations, while two SCM peaks existed at the eddy edge (i.e. S7), the upper SCM was around 50 m and the lower was around 100 m (Fig 2G and 2H) and the phenomena also observed at S8 (not sown in figures) in 2010. The integrated Chl *a* (I-Chl*a*, from surface to the depth of 200 m) did not display significant variation (23–33 mg m^−2^, Fig 3D) at all stations. High concentrations (67–103 mg m^−3^) of POC were shown at S7 and low values at S3 (43–69 mg m^−3^). In-situ incubation of PP at S3 and S7 showed a similar trend, with higher values near the surface and lower values close to the bottom of the euphotic zone (Fig 2G and 2H). The euphotic zone integrated PP (I-PP) at S3 (228 mg-C m^−2^ d^−1^), however, was lower than at S7 (373 mg-C m^−2^ d^−1^) (Fig 3E). The I-PP value at S3 was similar to the mean annual I-PP (285 mg-C m^−2^ d^−1^) in the main stream of the Kuroshio reported by Gong et al. (1999) [3].

**Fig 4:**
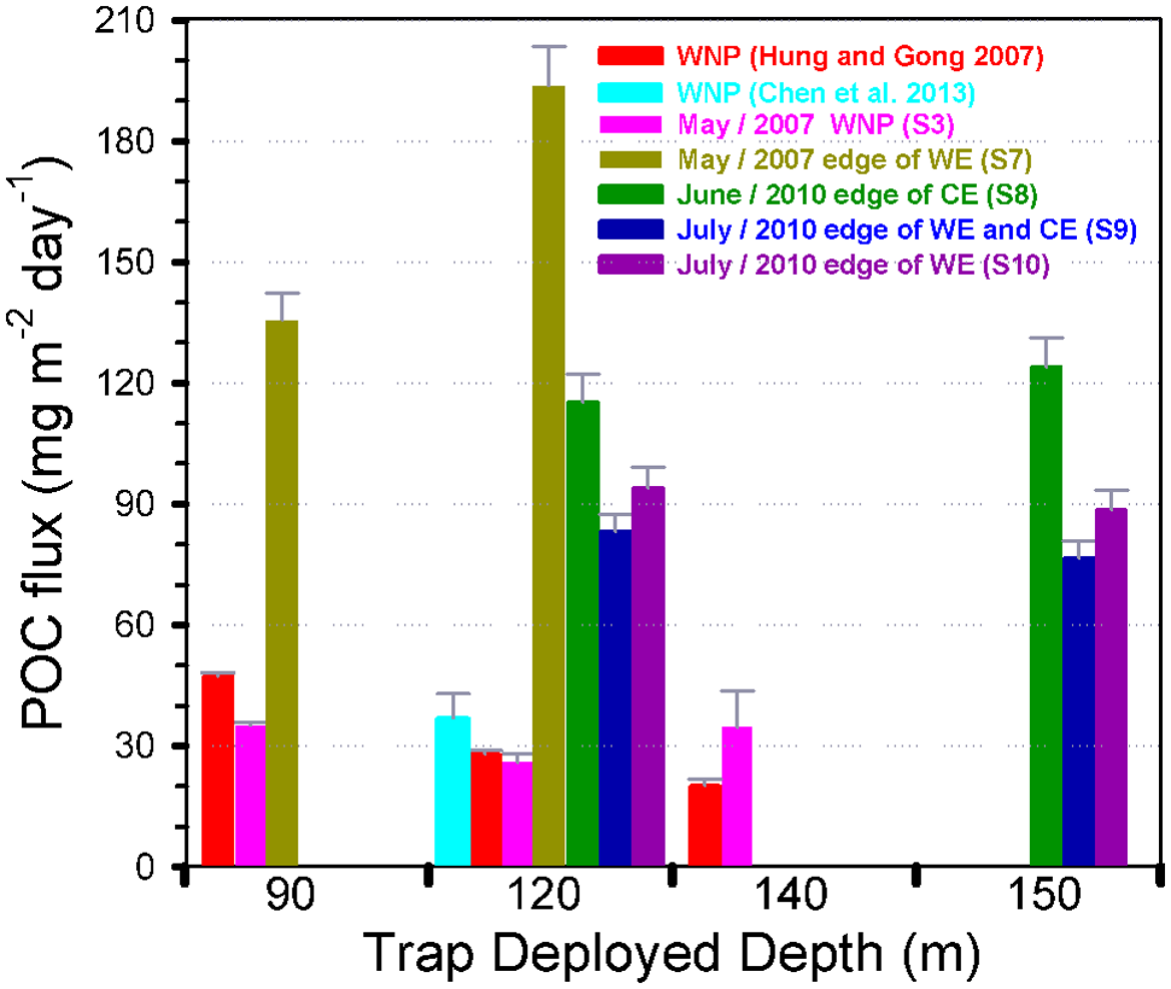
Comparison of POC fluxes in the WNP. POC fluxes below the bottom of the euphotic zone measured by floating intercepted sediment traps were compared among different studies in the WNP [4,35].

POC fluxes in both traps (unpoisoned and poisoned) at depths of 90, 120 and 140 m at S3 were 35, 26 and 35 mg-C m^−2^ d^−1^, and 43, 61 and 39 mg-C m^−2^ d^−1^ (Table 3, Fig 4), respectively. POC fluxes in unpoisoned traps at depths of 90 and 120 m at S7 were 136 and 194 mg-C m^−2^ d^−1^, respectively, and POC fluxes in poisoned traps at depths of 90 and 140 m were 232 and 211 mg-C m^−2^ d^−1^ (Table 3), respectively. POC fluxes in unpoisoned and poisoned traps at S8, S9 and S10 at depths of 120 and 150 m ranged from 115 to 124, 83 to 77, and 94 to 89 mg-C m^−2^ d^−1^, and from 195 to 211, 116 to 129, and 133 to108 mg-C m^−2^ d^−1^ (Table 3), respectively. The POC fluxes in the poisoned traps seems to be higher than those in the unpoisoned traps, revealing that increased POC flux could be from the release of POC into the poisoned traps by defecating swimmers ([29] and references therein). In order to compare our POC flux data to other reports (fluxes usually measured at 120 m) around the world, we adopted POC fluxes in unpoisoned traps at 120 m herein.

**Table 3:** Fluxes of POC (mg m^-2^ d^-1^) measured by floating intercepted sediment traps and ratios of (POC flux / I-PP) at the depth of 120 m in the Western North Pacific.

Station	POC_90m_	POC_120m_	POC_140m_	POC_150m_	I-PP_modeled_(mg m^-2^ d^-1^)	*e* ratio(POC flux / I-PP)
WNP(Hung and Gong 2007)	47±1	28±1	20±2	–	*430/252	0.06/0.11
^1^WNP(Hung et al. 2009)	–	71±16(^##^37 ± 6)	–	–	**285	0.25
^2^WNP(Chen et al. 2013)	–	52±13(^##^37±6)	–	–	**285	0.18
S3(Kuroshio)	35±2(^#^43±2)	26±2(^#^61±3)	35±9(^#^39±2)	–	*228/275	0.11/0.09
S7(edge of WE)	136±7(^#^232±12)	194±10	(^#^211±11)	–	*373/220	0.53/0.88
S8(edge of CE)	–	115±7(^#^195±11)	–	124±7(^#^211±7)	197	0.59
S9(edge of WE & CE)	–	83±4(^#^116±6)	–	77±4(^#^129±8)	159	0.52
S10(edge of WE)	–	94±5(^#^133±9)	–	89±5(^#^108 ± 6)	218	0.43

^1^: within Asian dust storm affected seasons [8];

^2^: with extreme weather events (i.e. Asian dust storms, typhoons and strong wind) [4];–: no data available;

^#^: POC preserved in brine and poison during collection period;

^##^: POC fluxes collected without EWEs [4];

*: in-situ incubation PP;

**: I-PP data derived from [3].

In comparison, the POC flux at S3 (26 mg-C m^−2^ d^−1^ at 120 m) is similar to the previously reported data (28 mg-C m^−2^ d^−1^ at 120 m) in the Kuroshio water by Hung and Gong (2007) [35] and in the oligotrophic waters (37 ± 6 mg-C m^−2^ d^−1^ at 120 m) in the WNP without extreme atmospheric events [4,8]. Remarkably, elevated POC fluxes (194 mg-C m^−2^ d^−1^ at S7, 115 mg-C m^−2^ d^−1^ at S8, 83 mg-C m^−2^ d^−1^ at S9, 94 mg-C m^−2^ d^−1^ at S10, Fig 4) at the eddy edges of mesoscale eddies in this study are much higher than the reference location and previously reported values in the oligotrophic water [4,8] (Table 3).

### Higher Particulate Organic Carbon Export Fluxes at the Eddy Edges

Since the POC export fluxes evaluated both by sediment trap-collected and ^234^Th/^238^U disequilibrium method are comparable in the subtropical oligotrophic ocean [23,24,35,36], any possible bias in POC fluxes measured in our study area should likely be insignificant. Here, we propose several mechanisms to interpret the high POC flux at the eddy edges. The sampling time was approximately the 95^th^ in warm eddy’s ~135 days life cycle which was based on the successive images of SSHA from CCAR. The sampling period was associated with the warm eddy’s stage from maturing to decaying. During that stage, the dominance of entrained nutrients, supporting phytoplankton growth, might have shifted to predominance in the biological response, which would have resulted in a flux of dead phytoplankton cells sinking out of the euphotic zone [10].

**Fig 5:**
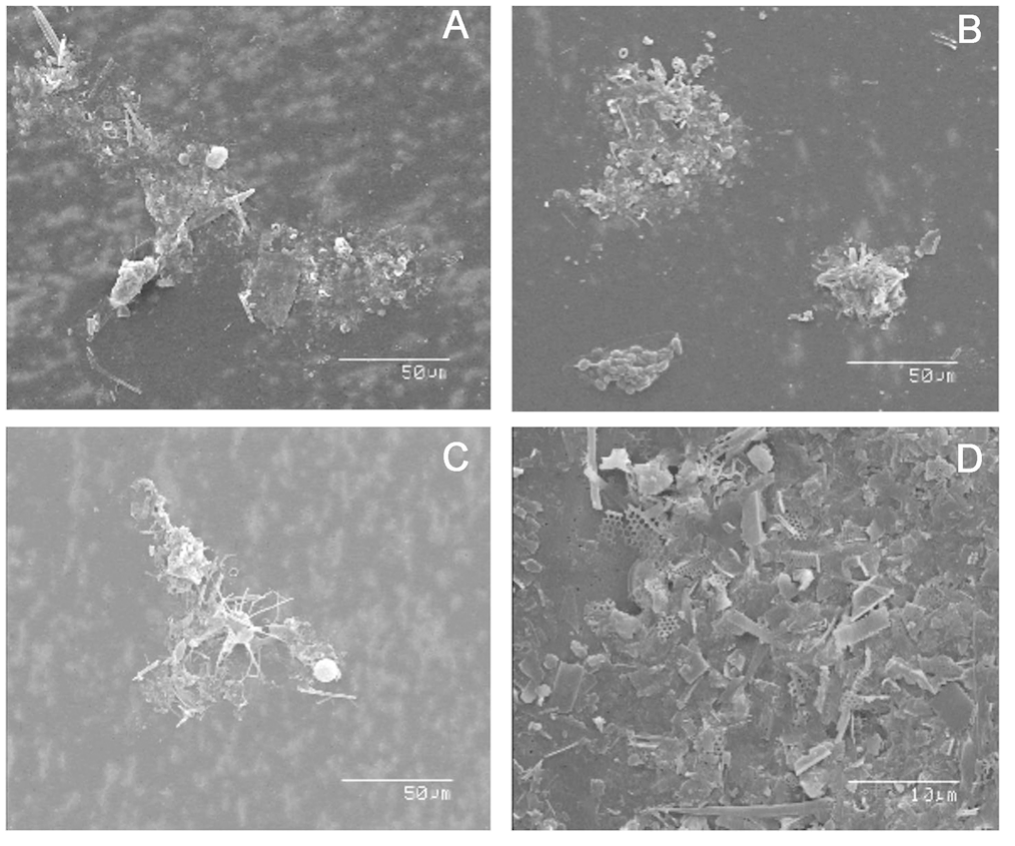
SEM images of bulk sinking particles at S9 and S10. SEM images of bulk sinking particles collected from sediment traps on Jul. 21, 2010 at the depth of 120m (A, B) and 150m (C, D) of S9 and S10, respectively. The composition of sinking particles are mainly fecal pellets, dead phytoplankton cells (e.g. diatom cells) and aggregates. Scale bars are 50 μm in panels A, B and C, and 10 μm in D.

Two Chl *a* peaks were observed at S6 and S7. Usually, one SCM is observed in the oligotrophic WNP, and the SCM might be controlled by the input of nutrients from below, supplied by turbulent mixing or upwelling [42,43], and light intensity. However, the surface layer (0–50 m) at S7 was oligotrophic with deeper D_ni_ (Table 2) than S3, suggesting a nitrogen limited region. According to Chen et al. (2007) [42], the calm, warm, and nitrate-depleted region is favorable for *Trichodesmium spp*. growth, especially in surface water. Chen et al. (2007) [42] also reported that *Trichodesmium spp*. can release other nitrogen sources (e.g. dissolved organic nitrogen, ammonium) as nutrients for other phytoplankton, such as diatoms in the upper SCM. While we did not examine the main phytoplankton species composition in the water column or in sinking particles in 2007, we did examine images of sinking particles at S9 and S10 in 2010. The main composition of sinking particles included diatoms (*nitzschia*, *navicula*. etc.), fecal pellets and aggregates of small particles (Fig 5). Chen et al. (2003, 2007) [42,40] and Hung et al. (2012) [31] reported that the deep SCM in the WNP and its adjacent areas are dominated by picoplankton assemblages. Although the sampling locations in this study are different from previous locations, the water characteristics in these studies are oligotrophic with a deep D_ni_, warm and high salinity water. As a consequence, the lower SCM that was observed in the deep subsurface layer might have been dominated by picoplankton assemblages as reported by previous researchers [31,42,44].

From the vertical distributions and standing stocks of Chl *a* at S3 and S7, one can see that the variation of Chl *a* concentrations and I-Chl *a* only showed slight difference (S3 = 0.2–0.6 and S7 = 0.2–0.4 mg-Chl *a* m^−3^; S3:S7 = 33 vs. 22 mg-Chl *a* m^−2^, respectively, Figs 2 and 3). Although we did not identify the composition and size of phytoplankton at both stations, the slightly lower values of Chl *a* concentrations and standing stocks also confirmed directly our postulation that large phytoplankton at S7 was more abundant than S3, since Chl *a* of large-size phytoplankton tends to be lower than that of small-size phytoplankton [45,46]. Additionally, both profiles of Chl *a* and POC concentrations at S7 depth of 120 m was still higher than 0.3 mg m^−3^ and 60 mg-C m^−3^, which were not close to background level (Chl *a* <0.1 mg m^−3^), respectively, suggesting that the anticycling eddy can carry particle down to depth.

The e-ratios (unpoisoned POC flux at 120 m / I-PP-modeled) of the reference station (S3) and edges of eddies (S7–S10) were 0.09–0.11 and 0.43–0.88, respectively (Table 3). The former is similar to the previously reported value (0.1) by [35], while the latter e-ratio is even higher than in the upwelling region (~0.2) near the northeast tip off Taiwan [47]. The reason for the low ratio of the POC export flux at S3 could be that the Kuroshio Current is too swift (5m ADCP records: 1–2 m s^−1^), which is not suitable for zooplankton grazing, compared to the prevailing slow currents (5 m ADCP records: 0.4–0.6 m s^−1^) at S7. Additionally, our in situ incubation PP (228–430 mg-C m^−2^ d^−1^) is significantly higher than the modeled PP (159–275 mg-C m^−2^ d^−1^) suggesting that some of PP might be underestimated in this study. As a consequence, the e-ratio should be lower than about 59% if we use in situ-PP. Alternatively, fecal pellets generated by zooplankton, appear to be important at S7; we found an abundance of fecal pellets when we picked and removed small swimmers under a microscope. Furthermore, SEM images of bulk sinking particles at S9 and S10 contained many fecal pellets (Fig 5), dead phytoplankton cells (diatoms, e.g. *nitzschia*, *navicula*, Fig 5A; *bacteriastrum*, Fig 5C; *coscinodiscus*, Fig 5D) and small aggregates, which strongly supports our contention that the enhanced POC flux could be caused by multiple factors such as elevated grazing by zooplankton (i.e. high abundance of fecal pellets), particle aggregation and phytoplankton metabolism.

Even though the discrepancy of I-PP differed from the *in-situ* incubation method and the modeled result, it could be attributed to the bias of adoptive optimal specific primary productivity [27,48], results showed that a high in-situ incubation PP value and an increased POC flux occurred simultaneously at the eddy edges of the mesoscale eddies (S7–S10). In other words, edges of eddies can significantly enhance the POC export from the euphotic zone owing to increased zooplankton grazing and possibly calm oceanic conditions. The latter postulation would need more field investigations for validation.

### Comparison of Particulate Organic Carbon Export Fluxes in Different Eddy Systems

**Fig 6:**
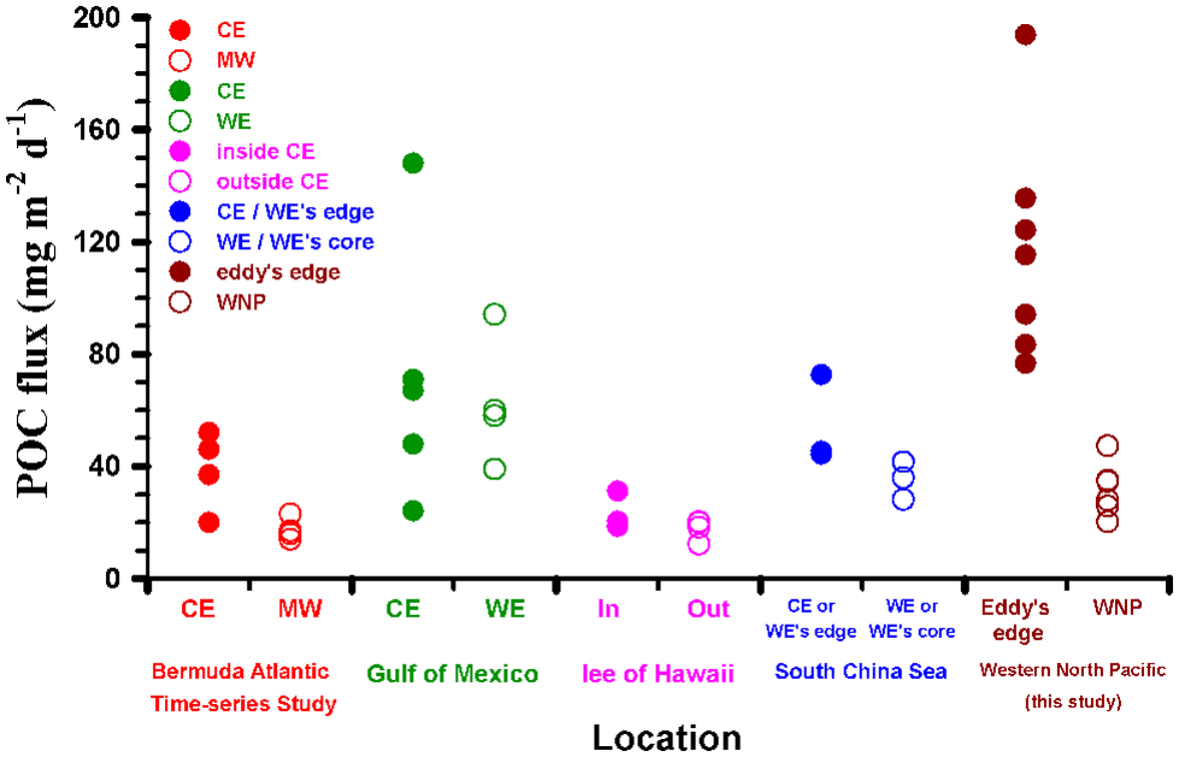
Comparison of POC export fluxes in different regions. Comparisons of POC fluxes related to the effect of mesoscale eddies which were reported in different study areas by trap-collected or ^234^Th / ^238^U disequilibrium or modeled methods. Data are derived from the references in the Table 1 and this study. Solid and empty symbols represented eddy edge and WNP stations, respectively.

The sinking POC export fluxes (77–194 mg-C m^−2^ d^−1^) at the eddy edges are significantly higher (a 2~7 fold) than fluxes determined by sediment traps from the reference location (i.e. S3: 26–35 mg-C m^−2^ d^−1^) and oligotrophic water in the WNP without and within extreme weather events (EWEs) (37 ± 6 and 52–71 mg-C m^−2^ d^−1^) [4,8], but are comparable to values measured in eddy system reported by Hung et al. (2004, 2010a) [19,24]. Even we compare our POC fluxes to other fluxes at different mesoscale eddy systems around the world (Table 1, those POC fluxes were estimated by either sediment trap or ^234^Th /^238^U disequilibrium methods, as well as model calculation [49]), one will see higher POC fluxes occurring in the WNP (Fig 6). Although Zhou et al. (2013) [25] estimated POC fluxes across several eddies (including eddy cores and edges) and found higher POC fluxes at the eddy edges in the South China Sea, the estimated POC fluxes (42–45 mg-C m^−2^ d^−1^, based on ^234^Th/^238^U method) are still lower than the measured values in this study. Besides, the reported POC fluxes (12–33 mg-C m^−2^ d^−1^) in the lee of eddy near Hawaii in the subtropical Pacific Ocean are also lower than our observed values. The possible reasons might be due to following factors: 1) different methods (Zhou et al. (2013) [25] used Th-234 method with some assumptions) may have their analytical uncertainties. For example, Benitez-Nelson et al. (2007) [21] reported POC fluxes measured by traps, N-15 mass balance and Th-234 method, were 18, 33 and 12 mg-C m^−2^ d^−1^, respectively. It is noted that the lowest POC flux was obtained by Th-234 method. If we assume the ratio of two methods is 1.5 (trap/Th-234 method = 1.5), POC fluxes measured by traps in the SCS will be 63~68 mg-C m^−2^ d^−1^, which is slightly lower than our values (83–115 mg-C m^−2^ d^−1^) in 2010; 2) phytoplankton species shifts may be an important factor, as mentioned earlier, the main phytoplankton in sinking particle in this study composed of large denuded diatom frustules such as *nitzschia*, *navicula*, *bacteriastrum*, *coscinodiscus*, which could be different from other studies [19,21,25]; also the study area is not a silicate limitation region based on our field observation which is quite different from the central subtropical Pacific Ocean [21]; 3) temporal and spatial variation of POC flux may exist around mesoscale eddy edge and eddy core in both WNP and SCS; 4) As mentioned by Benitez-Nelson et al. (2007) [21], total organic carbon within the euphotic zone can be apparently degraded by bacterioplankton, in other words, strong microbial community, coupling of production, grazing and remineralization processes may restrain organic carbon export; 5) fecal pellets were found in this study, but it was a lack of fecal pellets in the lee of eddy near Hawaii [21]. It suggests that zooplankton grazing is an important mechanism to transport particle out of euphotic zone. Although we can rule out these facts, our study provides evidence that edges of mesoscale eddies in the WNP and in the Gulf of Mexico [19,24] have high carbon sequestration potential as compared to those in the subtropical Pacific Ocean—lee of Hawaii [20–22], the Sargasso Sea [23] and the South China Sea [24,45,49]. Overall, our new results support previous field observations that the edge of mesoscale eddies may be a key conduit to transport organic matter to the ocean interior [24,25]. However, this hypothesis needs more intensive and detailed field observations to prove it.

The biogenic carbon flux to the deep ocean is one of the main factors affecting CO_2_ partial pressure in the atmosphere. Mesoscale eddies have a notable impact on POC flux, but how important are they to the POC flux in the WNP. We briefly estimate the POC flux based on the eddy distribution in the WNP from 21°N to 26°N and 121°E to 125°E if we assume most eddies in the WNP have similar carbon sequestration rate. POC fluxes at the eddy edge and reference site are assumed 110 and 30 mg-C m^−2^ d^−1^, respectively. If 3 mesoscale eddies are formed each year, and the total high POC flux period is 30 days (10 days x 3), the eddy induced POC flux will be 3300 mg-C m^−2^ y^−1^ (= 110 mg-C m^−2^ d^−1^ x 30 d y^−1^), and the reference POC flux will be 10050 mg-C m^−2^ y^−1^ (= 30 mg-C m^−2^ d^−1^ x 335 d y^−1^). This suggests that the contribution of eddy in the WNP can account for at least 20~30% of annual carbon export flux. As mentioned earlier, an eddy’s life cycle, nutrient supply and phytoplankton physiology are difficult to predict or model. This study thus provides important information on the POC flux along the eddy’s edge that is relatively high as compared to the reference region. More intensive observations are needed to further validate and document the natural episodes in the oligotrophic oceans.

## Conclusions

Based on three sea-going expeditions in 2007 and 2010, elevated organic carbon fluxes, a 2~4 fold higher than POC fluxes in similar oligotrophic waters, were observed at eddy edges in the oligotrophic waters of the Western North Pacific Ocean. Although we are unable to exactly elucidate which factors (fecal pellets, dead phytoplankton detritus and aggregates) mainly drive organic carbon flux in those eddies, it is clear that enhanced POC flux appearing at eddy edges reshape the picture that “inefficient POC export” did not always happen in the warm waters in the subtropical. Most importantly, POC flux in the oligotrophic waters in the oligotropical open ocean could be underestimated due to limited sampling at mesoscale eddy edges. Hence, the large scale measurement of POC flux in the oligotrophic waters should be conducted in the further study.

## Supporting Information

S1 File Hydrographic data in this study. Hydrographic data of density (kg m^−3^), temperature (°C), salinity and concentrations of nutrients (μM, including N, P and Si) and Chl *a* (mg m^−3^) are within the supporting information file.
(XLSX)
